# Thaumatin-like proteins and a cysteine protease inhibitor secreted by the pine wood nematode *Bursaphelenchus xylophilus* induce cell death in *Nicotiana benthamiana*

**DOI:** 10.1371/journal.pone.0241613

**Published:** 2020-10-30

**Authors:** Haru Kirino, Kohki Yoshimoto, Ryoji Shinya

**Affiliations:** 1 School of Agriculture, Meiji University, Kawasaki-shi, Kanagawa, Japan; 2 JST PRESTO, Kanagawa, Japan; National Institute of Technology Rourkela, INDIA

## Abstract

Pine wilt disease (PWD) is an infectious disease of pines that typically kills affected trees. The causal pathogen of PWD is the pine wood nematode (PWN), *Bursaphelenchus xylophilus*. Understanding of the disease has advanced in recent years through the use of a highly sensitive proteomics procedure and whole genome sequence analysis; in combination, these approaches have enabled identification of proteins secreted by PWNs. However, the roles of these proteins during the onset of parasitism have not yet been elucidated. In this study, we used a leaf-disk assay based on transient overexpression in *Nicotiana benthamiana* to allow functional screening of 10 candidate pathogenic proteins secreted by PWNs. These proteins were selected based on previous secretome and RNA-seq analyses. We found that five molecules induced significant cell death in tobacco plants relative to a GFP-only control. Three of these proteins (Bx-TH1, Bx-TH2, and Bx-CPI) may have a role in molecular mimicry and likely make important contributions to inducing hypersensitive responses in host plants.

## Introduction

Pine wilt disease (PWD) is an infectious disease of pine trees that typically kills affected trees. PWD has caused serious forest damage in East Asian countries for more than 100 years; it spread to European countries at the end of the twentieth century [1–4]. PWD is currently recognized as one of the most serious tree diseases worldwide [5,6]. The causal pathogen is the pine wood nematode (PWN), *Bursaphelenchus xylophilus* [1]. The PWN has phytophagous and mycophagous phases during its life cycle, and is transmitted by vector pine sawyer beetles of the genus *Monochamus* [7,8].

The mechanisms of PWD have been extensively studied worldwide over the past 50 years. There is now considerable knowledge regarding the physiological and histological reactions of pine trees against PWNs during infection, and the disease process (i.e., from nematode infection to pine death) has been closely observed [9,10]. Based on observational studies, Myers [11] and Futai [12] postulated that the spread of the PWN through a tree causes a series of hypersensitive responses that eventually lead to the death of susceptible pines. The molecular biological reactions between PWNs and pine trees during PWD were poorly understood until recent investigations were initiated. Hirao et al. [13] compared gene expression patterns between resistant and susceptible pine trees after PWN infection and found that the expression levels of pathogenesis-related genes (e.g., PR-1b, 2, 3, 4, 5, 6) were much higher in susceptible trees inoculated with virulent nematodes than in resistant trees at all time points. This finding provided evidence at the molecular level that fully supported the postulate of Myers [11] and Futai [12].

Because an excessive defense response of pines against PWNs leads to tree death, it has been suggested that the pathogenicity (or virulence) of the PWN is dependent on the ability of the nematodes to avoid host defense responses (e.g., through production of reactive oxygen species or phytoalexin) and/or disturb the defense responses of the trees [14]. The antioxidant activity of the PWN has been studied recently; the activity levels of antioxidant molecules (i.e., glutathione s-transferase, catalase, and peroxiredoxin) were higher in virulent isolates of the PWN than in avirulent isolates [15–17]. Although the existence of molecules that induce an excessive defense response has been suggested (e.g., cell-wall degrading enzymes and venom-allergen protein) [18–27], no molecule has yet been identified as the pathogenicity factor that induces or disturbs the host defense response by establishing a technical barrier. The available tools for molecular functional analysis of PWNs and pine trees remain limited. RNA interference (RNAi) [28], CRISPR/Cas9 [29], and transgenesis techniques [30] have been established and widely employed for molecular functional studies of the free-living nematode, *Caenorhabditis elegans*. However, CRISPR/Cas9 and transgenesis techniques have not been developed for PWNs, and RNAi is insufficiently robust for pathogenicity testing against pine trees [31]. Presently, there are no techniques for producing transgenic Japanese black pines (*Pinus thunbergii*) or Japanese red pines (*P*. *densiflora*).

In this study, we used a leaf-disk assay based on transient overexpression in *Nicotiana benthamiana* to facilitate functional screening of 10 candidate PWN pathogenic molecules suspected to be involved in PWN parasitism and/or PWD. The candidate molecules are listed in Table 1. The first group of candidate molecules comprises plant-like proteins that have relatively high sequence similarity to the pathogenesis-related protein of plants. Secretome analysis in our previous investigations showed that the PWN secretes three plant-like proteins: plant thaumatin-like 1 (Bx-TH1; BUX.s 00036.89), plant thaumatin-like 2 (Bx-TH2; BUX.s 00036.92), and plant cysteine protease inhibitor-like (Bx-CPI; BUX.s 00351.347) [18]. The second group comprises molecules that are highly expressed when nematodes feed on plants cells at the early stage of PWN infection, but are poorly expressed in the mycophagous stage. In this study, we selected seven of these proteins based on a previous transcriptome analysis of the PWN [32]: unknown proteins (Bx-NA1; BUX.s 00083.48, Bx-NA2; BUX.s 01662.77), venom allergen-like protein vap3 (Bx-VA; BUX.s 00116.606), aspartic protease (Bx-AP; BUX.s 00713.953), cysteine protease 1 (Bx-CP1; BUX.s 01147.176), cysteine protease 2 (Bx-CP2; BUX.s 01147.177), and glutathione s-transferase protein (Bx-GST; BUX.s 01254.333). All of these proteins are reportedly abundant in molecules secreted by the PWN [18]. We aimed to determine whether these candidate molecules could induce cell death in tobacco plants.

**Table 1:** List of candidate proteins.

GeneDB accession	Putative functional annotation	Protein name	Reference
BUX.s00036.89	Plant thaumatin-like	Bx-TH1	Shinya et al. [18]
BUX.s00036.92	Plant thaumatin-like	Bx-TH2	
BUX.s00351.347	Plant cysteine protease inhibitor-like	Bx-CPI	
BUX.s00083.48	---NA---	Bx-NA1	Espada et al. [32]
BUX.s00116.606	Venom allergen-like protein vap3	Bx-VA	
BUX.s00713.953	Aspartic protease	Bx-AP	
BUX.s01147.176	Cysteine protease	Bx-CP1	
BUX.s01147.177	Cysteine protease	Bx-CP2	
BUX.s01254.333	Glutathione s-transferase protein	Bx-GST	
BUX.s01662.77	---NA---	Bx-NA2	

NA, not available.

## Results

### Three proteins secreted by the PWN induced plant cell death

**Fig 1:**
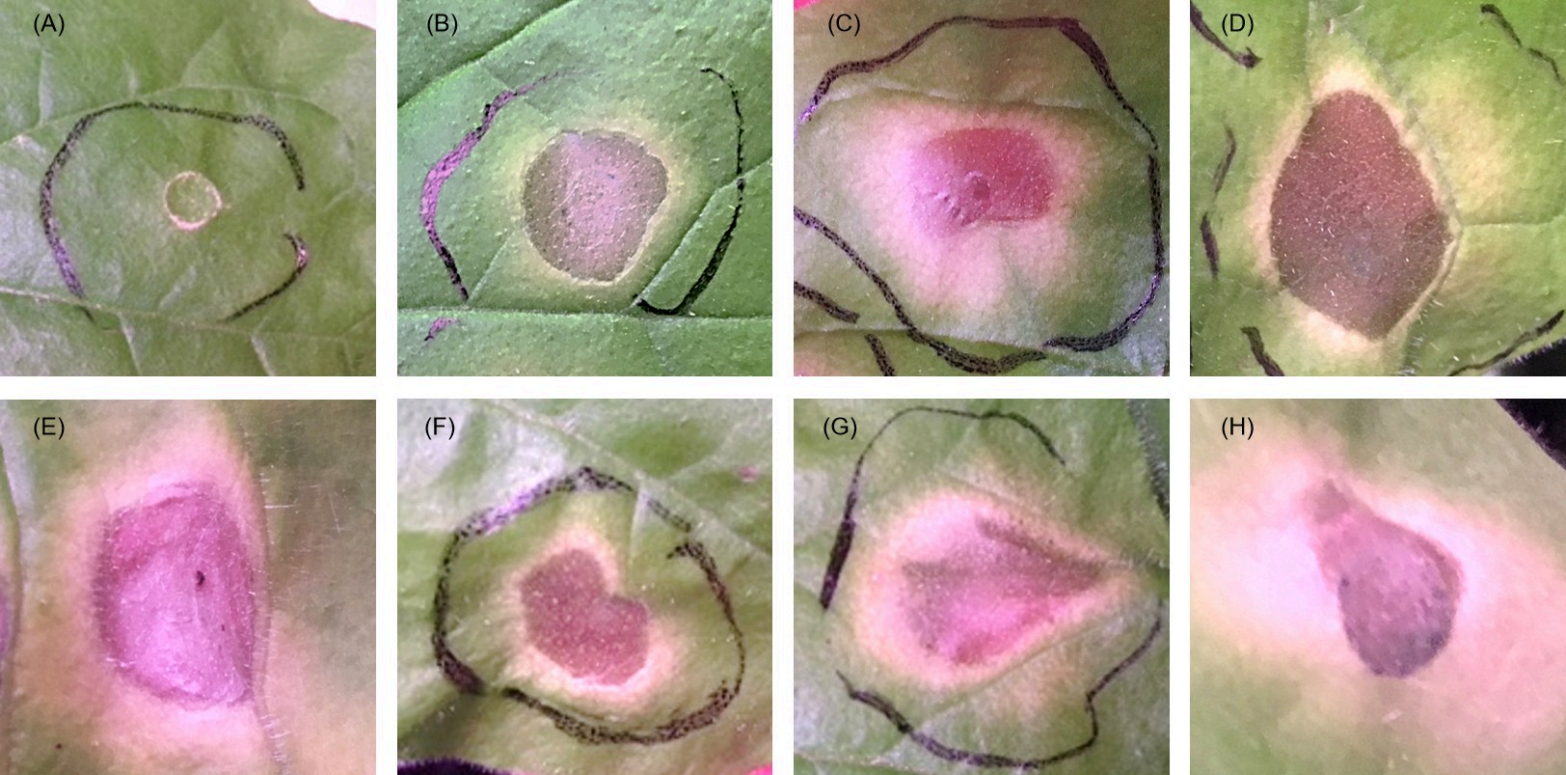
Symptoms of cell death induced by pathogenic candidate proteins. Leaves of *Nicotiana benthamiana* were infiltrated with *Agrobacterium tumefaciens* strains to express candidate *Bursaphelenchus xylophilus* proteins. (A) The GFP-only vector control. (B) Bx-TH1 with SP, (C) Bx-TH1 without SP, (D) Bx-TH2 with SP, (E) Bx-TH2 without SP, (F) Bx-CPI with SP, (G) Bx-NA1 with SP, and (H) Bx-VA without SP all induced significant cell death relative to a GFP-only control. Images were captured 10 days after infiltration. SP, secretory signal peptide.

**Fig 2:**
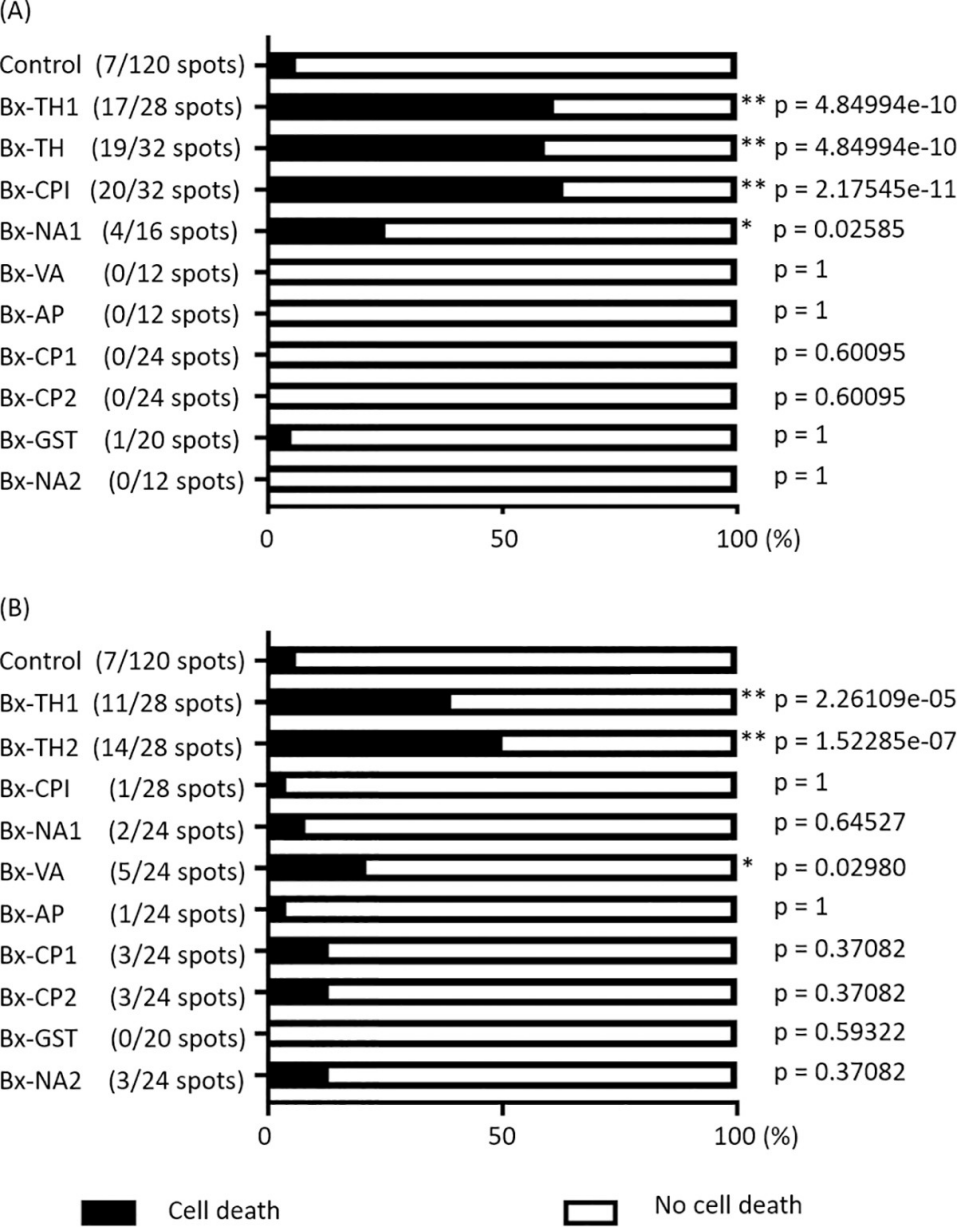
Rates of cell death induced by the candidate pathogenic proteins. The rates of cell death induced by candidate molecules with (A) and without SPs (B) are presented graphically. The degree of symptoms after infiltration was monitored visually and categorized into two classes: Cell death (black bar) and no cell death (white bar). The proportions of cell death in parentheses were analyzed statistically using Fisher’s exact test (** p < 0.01, * p < 0.05). Asterisks indicate a significant increase in cell death induced by each of the 10 candidate pathogenic proteins compared with the GFP-only vector control. SP, secretory signal peptide.

Using pUBQ10 promoter control, we generated transgenic *N*. *benthamiana* that expressed pathogenicity candidate molecules (Table 1) with or without the putative secretory signal peptide (SP), and investigated the function of these molecules in tobacco plant cell death (Figs 1 and 2). Two plant thaumatin-like proteins, Bx-TH1 (BUX.s00036.89) and Bx-TH2 (BUX.s00036.92), induced statistically significant cell death, regardless of the presence of putative SPs relative to a GFP-only control (p < 0.01). A plant cysteine protease inhibitor-like protein, Bx-CPI (BUX.s00351.347), induced significant cell death only when the sequence contained a putative SP relative to a GFP-only control (p < 0.01). An unknown protein, Bx-NA1 (BUX.s 00083.48), with SP, and a venom allergen-like protein, Bx-VA (BUX.s 00116.606), without SP, induced significant cell death relative to a GFP-only control (p < 0.05), although the probability of inducing cell death by Bx-NA1 and Bx-VA was lower than that for the three plant-like proteins. When putative SPs were inserted into the 10 sequences, no significant cell death relative to a GFP-only control occurred in relation to the other proteins included in this study: Bx-AP (BUX.s 00713.953), Bx-CP1 (BUX.s 01147.176), Bx-CP2 (BUX.s01147.177), Bx-GST (BUX.s01254.333), or Bx-NA2 (BUX.s01662.77).

### Protein abundance was not related to cell death

**Fig 3:**
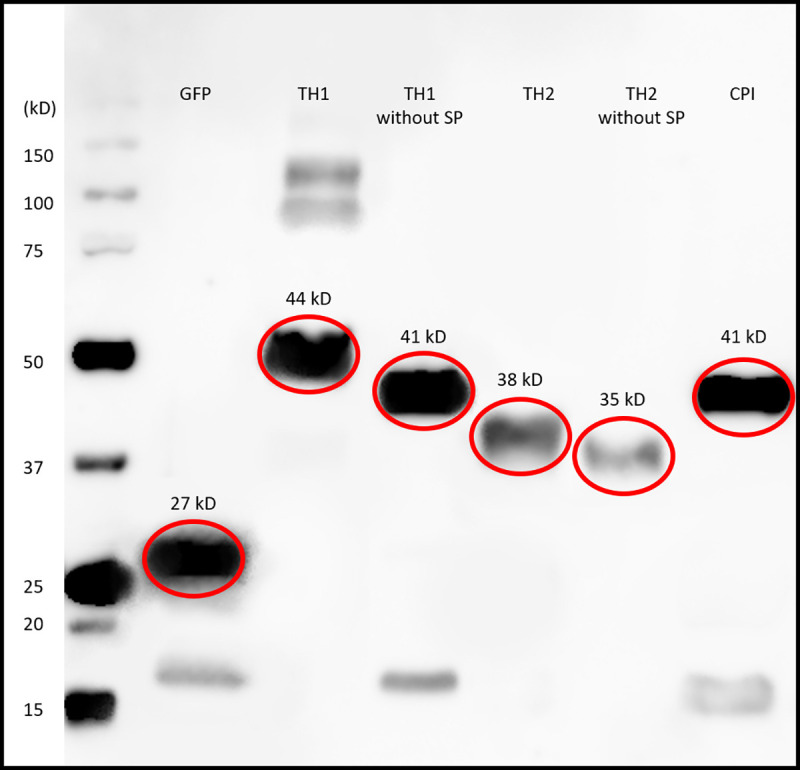
Bands of plant-like proteins on Western blot membranes. The quantities of the candidate proteins expressed in *Nicotiana benthamiana* were determined by Western blot analysis. GFP (control protein) and GFP fusion candidate proteins were detected with anti-GFP antibodies. The protein bands of Bx-TH1 with SP (44 kDa) and without SP (41 kDa), Bx-TH2 with SP (38 kDa) and without SP (35 kDa), and Bx-CPI with SP (41 kDa) are shown on the membrane. The GFP control band is approximately 27 kDa. SP, secretory signal peptide.

Using Western blot analysis, we examined three plant-like proteins (Bx-TH1, Bx-TH2, and Bx-CPI) that induced significant cell death in *N*. *benthamiana* relative to a GFP-only control (p < 0.01). This analysis allowed measurement of the amount of each protein produced, compared with the GFP-only vector control. The genes of three plant-like proteins were fused to the gene encoding green fluorescent protein (GFP) at the C-terminus by inserting cDNAs into the vector pUBC-GFP-Dest, which was then transfected into *N*. *benthamiana*. Three days after transfection, the fusion proteins were extracted and analyzed by Western blot analysis using anti-GFP polyclonal antibodies, which showed that the amounts of Bx-TH1 with and without the SP and Bx-CPI with the SP had values similar to the GFP-only vector control (Fig 3). The amounts of all other proteins were less than in the GFP-only vector control. Therefore, the abundances of the three plant-like proteins were not related to cell death. In Fig 3, a few weak bands were observed in the GFP-only vector control, Bx-TH1 without SP, and CPI. Since these bands were all about 17 kDa, they are probably non-specific bands due to the secondary antibody. The extra bands (about 90 and 130 kDa) in TH1 with SP may be a dimer and trimer.

### The intracellular localization of Bx-TH1, Bx-TH2, and Bx-CPI

**Fig 4:**
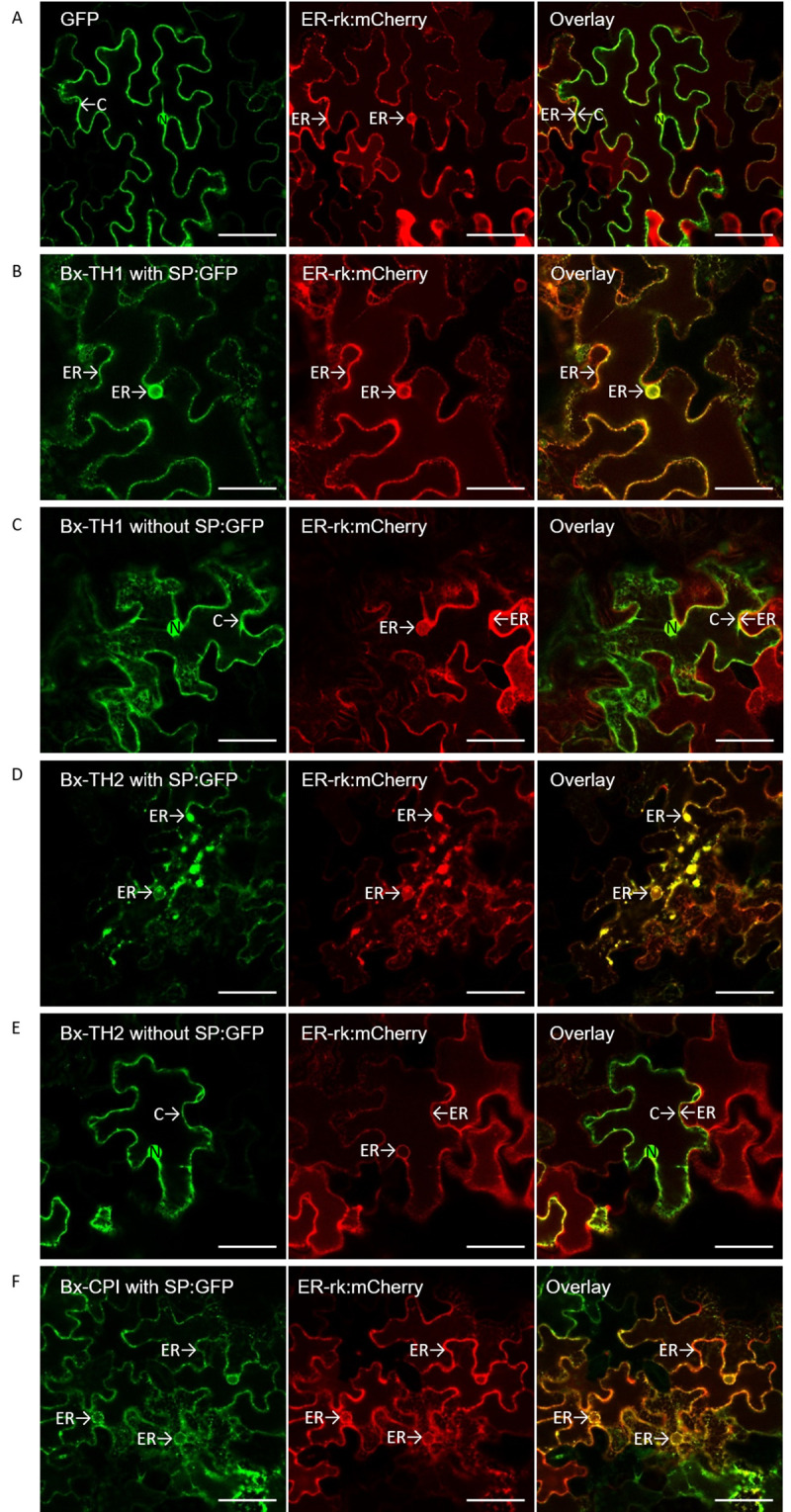
Localization of plant-like proteins in plant cells. Confocal images of (A) GFP, (B) Bx-TH1 with SP:GFP, (C) Bx-TH1 without SP:GFP, (D) Bx-TH2 with SP:GFP, (E) Bx-TH2 without SP:GFP, and (F) Bx-CPI with SP:GFP infiltrated into *Nicotiana benthamiana* leaves together with an mCherry-labeled endoplasmic reticulum marker (ER-rk CD3-959). For confocal laser scanning microscopy, samples were taken 3 days postinoculation, and fluorescent channels were scanned sequentially. GFP fluorescence is shown in green (left panel) and mCherry fluorescence in red (middle panel). Overlay of green and red signals appear yellow (right panel). N, nucleus; C, cytoplasm; ER, endoplasmic reticulum; SP, secretory signal peptide. Scale bar = 50 μm.

To determine the subcellular localization of the three plant-like proteins, Bx-TH1, Bx-TH2, and Bx-CPI were fused to the gene encoding GFP then transfected into *N*. *benthamiana* with an mCherry-labeled endoplasmic reticulum marker (ER-rk CD3–959). Visualization of transiently expressed GFP fusion proteins and mCherry-labeled endoplasmic reticulum marker proteins in tobacco leaves was performed by confocal microscopy (Fig 4). The GFP signals of Bx-TH1, Bx-TH2, and Bx-CPI with SPs were co-localized with the endoplasmic reticulum marker (Fig 4B, 4D and 4F), whereas the GFP signal of the vector control was present in both the plant cytoplasm and nucleus (Fig 4A). The GFP signals of Bx-TH1 and Bx-TH2 without SPs were found in both the plant cytoplasm and nucleus, similar to the localization of the GFP signal of the vector control.

### *Bx-TH1* and *Bx-CPI* mRNAs were expressed in the intestine of adult *B*. *xylophilus*

**Fig 5:**
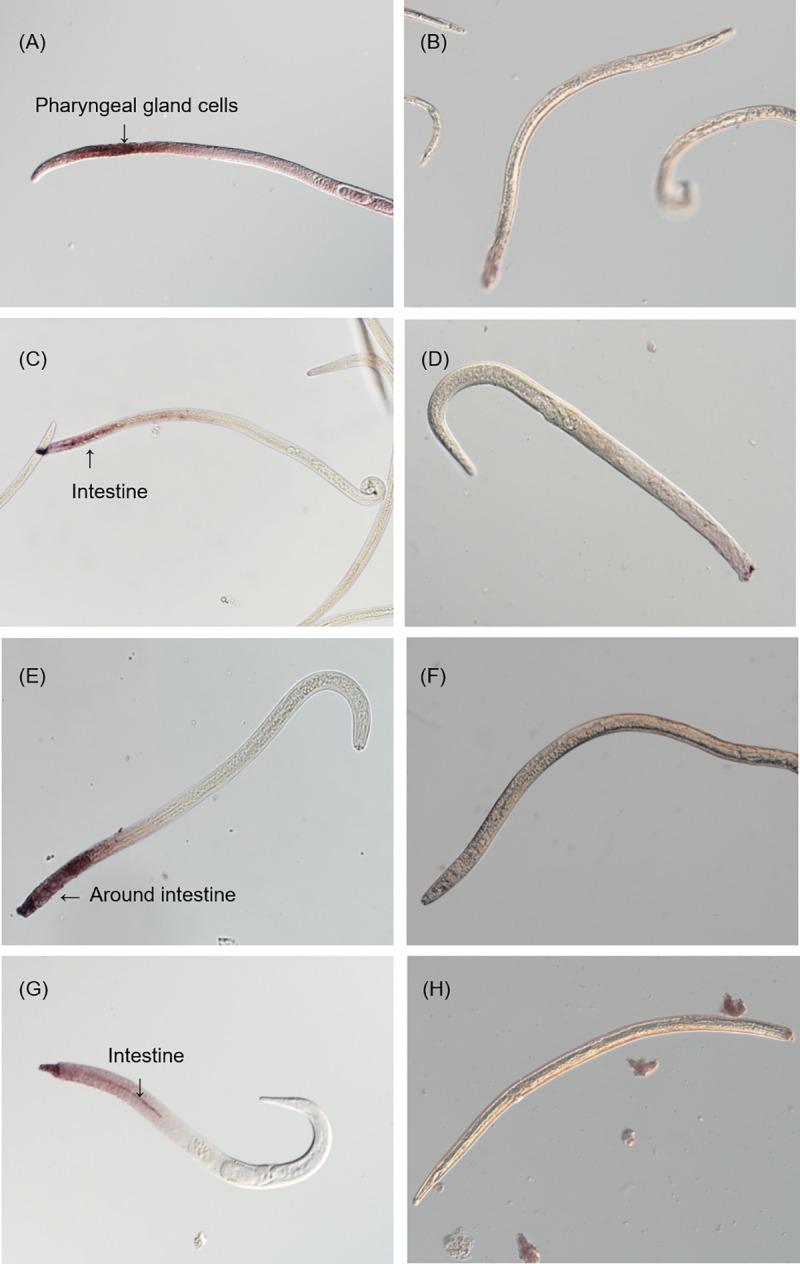
The mRNA localization of *Bx-TH1*, *Bx-TH2*, and *Bx-CPI* in nematodes. The localization of *Bx-ENG-1* [19] (A) as a positive control for gene expression in pharyngeal gland cells as shown by *in situ* hybridization. Localization of transcripts encoding plant-like proteins of the pine wood nematode in the intestine as shown by *in situ* hybridization, except for the sense probes of *Bx-ENG-1* (B), *Bx-TH1* (D), *Bx-TH2* (F), and *Bx-CPI* (H), which produced no signal. (C) *Bx-TH1*, (E) *Bx-TH2*, and (G) *Bx-CPI*.

*In situ* mRNA hybridization was used to investigate the spatial expression patterns of the three plant-like proteins in adult stage of the PWN. *Bx-TH1* and *Bx-CPI* were expressed in the intestine of adult *B*. *xylophilus* (Fig 5C and 5G, S1 Fig). Similar signal patterns were detected in both female and male nematodes. Digoxigenin-labeled antisense probes generated from the cellulase gene (*Bx-ENG-1*), which is reportedly expressed in the esophageal gland cell [19], were used as a positive control. The signal of *Bx-ENG-1* was detected specifically in the esophageal gland cell (Fig 5A). Although a diffuse signal was observed for *Bx-TH2* around the intestine (Fig 5E), no clear localization was obtained reproducibly in a specific area. No hybridization with the control sense cDNA probes was observed in the nematodes (Fig 5B, 5D, 5F and 5H).

## Discussion

We conducted an *in planta* functional analysis of 10 candidate pathogenicity proteins secreted by the PWN; we used *N*. *benthamiana* to screen for molecules that induced hypersensitive cell death in tobacco. Three candidate molecules (two thaumatin-like proteins [Bx-TH1 and Bx-TH2] and a cysteine protease inhibitor [Bx-CPI]) induced significant cell death in *N*. *benthamiana* relative to a GFP-only control (Figs 1 and 2). All of these proteins have putative SPs and have been shown to be abundantly secreted by *B*. *xylophilus*, according to mass spectrometry-based proteome analysis [18].

Pathogenesis-related protein 5 (PR-5) has been identified as thaumatin, a pathogenesis-related gene associated with systemic acquired resistance; it is present in many organisms, including plants [33,34], nematodes [35], insects [36], and fungi [37,38]. The amino acid sequence of Bx-TH1 shares 32% identity with the thaumatin-like protein of *Pinus monticola* (ADB97933.1) [39], though no highly similar sequence (e-value < 1e^-10^) was found in all organisms. Meng et al. (2020) [40] reported that Bx-TH2 is similar to the Hall’s panicgrass *Panicum hallii* thaumatin-like protein (XP_025808117.1), and it shares 52.05% identity with this protein, with over 85% query coverage. In plants, thaumatin is induced by a variety of phytopathogens via salicylic acid [33,34]. Transgenic wheat and tobacco lines constitutively expressing rice and groundnut thaumatin-like genes, respectively, exhibit enhanced resistance to the fungal pathogens *Fusarium graminearum* and *Rhizoctonia solani*, respectively [41,42]. Furthermore, thaumatin in these plants may have a role in activating other plant defense pathways, including the production of phenylpropanoid and phytoalexin [43]. Silencing *Bx-TH1* by RNAi inhibited the ability of PWNs to induce cavitation in pine trees after inoculation [44]. Using tobacco plants, we conducted functional analyses of both Bx-TH1 and Bx-TH2 and clearly showed that both molecules could induce hypersensitive cell death in these plants. Further investigation is necessary to clarify whether Bx-TH1 and Bx-TH2 induce hypersensitive cell death in host pines.

Bx-CPI with SP induced hypersensitive cell death in our study. We previously reported that Bx-CPI is most similar to a cystatin-like peptidase inhibitor in the herbaceous plant *Medicago truncatula* [18]. However, Meng et al. (2020) [40] reported that Bx-CPI is more similar to the CP inhibitor five-like protein of the common sunflower *Helianthus annuus* (XP_022033148.1), which shares 32.5% identity with this protein, with over 62% query coverage based on a BLAST analysis of the current database. Although the phylogenetic analysis showed that Bx-CPI clustered in the same clade with an unnamed *Enterobius vermicularis* (Oxyuridae) protein, no other nematode proteins were clustered to the same group [40]. Cystatin mainly inhibits peptidases in the C1 (papain) and C13 (legumain) peptidase families; however, it has multiple roles, including the control of endogenous cysteine peptidases, seed development, and programmed cell death (in which it acts as both the regulator and executioner) [45–47]. Plant cystatins also have significant roles in plant defenses against pathogenic microbes, herbivorous arthropods, and nematodes that are parasitic in plants [46,48,49]. The number of peptidase inhibitors secreted by *B*. *xylophilus* is significantly greater than the numbers in the secretomes of *Meloidogyne incognita* and *Brugia malayi*, particularly with respect to cysteine peptidase inhibitors [18]. Although little is known regarding CPI in *B*. *xylophilus*, Xue and Wu (2019) [50] reported that a cysteine protease inhibitor is involved in the development of PWNs. Here, we demonstrated that a different cysteine protease inhibitor of PWNs can induce hypersensitive cell death in *N*. *benthamiana*.

Although the mechanisms by which Bx-TH1, Bx-TH2, and Bx-CPI cause hypersensitive cell death in tobacco remain unknown, these plant-like proteins were localized to the plant cytoplasm/apoplast, nucleus, and endoplasmic reticulum (Fig 4). The SP of each nematode secretory protein is generally expected to be cleaved off before secretion from the nematode stylet. However, we generated transgenic *N*. *benthamiana* that expressed candidate pathogenicity molecules with or without the putative SP to determine the effect of the SP on protein localization *in planta* and whether the PWN secretory proteins can be processed during translocation in plants. Bx-TH1 and Bx-TH2 constructs with and without SPs differed in intracellular localization and induced hypersensitive cell death irrespective of the presence of SPs. This suggests that Bx-TH1 and Bx-TH2 can cause hypersensitive cell death regardless of their intracellular localization. By contrast, Bx-CPI induced significant cell death only when the sequence contained a putative SP relative to a GFP-only control. When Bx-TH1 and Bx-TH2 were transiently expressed in *N*. *benthamiana* leaves, protein bands corresponding to Bx-TH1 with SP (44 kDa) and without SP (41 kDa) and to Bx-TH2 with SP (38 kDa) and without SP (35 kDa) were detected by Western blot analysis. The sizes of these protein bands matched the expected sizes, indicating that the putative SPs were not cleaved off *in planta*. There are two possible explanations for these results. First, the SPs of Bx-TH1 and Bx-TH2 are cleaved off before their delivery from the PWN into the host cell, and they are localized to the cytoplasm/nucleus in plant cells to induce cell death. Alternatively, the SPs are not cleaved off before delivery from the PWN to the host cell, and the proteins target the endoplasmic reticulum via the SP. The Bx-CPI without SP was localized to the endoplasmic reticulum as was Bx-CPI with SP (data not shown). Therefore, the putative SP in Bx-CPI may be involved in the protein’s structure. Additional biochemical experiments are necessary to understand the actual role of the SPs in Bx-TH1, Bx-TH2, and Bx-CPI. Given that an endoplasmic reticulum stress-inducible protein was up-regulated in PWN-infested *P*. *pinaster* (a pine species very susceptible to PWN) when compared with PWN-infested *P*. *pinea* (less susceptible pine species) [51], Bx-TH1, Bx-TH2, and Bx-CPI localized to the endoplasmic reticulum may induce hypersensitive cell death in pines and have an important role in pine wilt disease. Since in situ hybridization analysis showed that *Bx-TH1* and *Bx-CPI* were transcribed in the intestines and *Bx-TH2* was expressed around the intestine of PWNs (Fig 5), it is also necessary to clarify how nematodes secrete these proteins and to determine their in vivo localization.

It has been suggested that Bx-TH1, Bx-TH2, and Bx-CPI mimic host plant defense systems [18]. Although a very high level of similarity in the amino acid sequences between these PWN proteins and plant proteins was not observed, Wang et al. [39] and Meng et al. [40] suggested that the possible role of these PWN proteins is to mimic host pine defense systems based on the result of phylogenetic and structure prediction analyses. Molecular mimicry is among the best-known strategies for host–parasite defense system evasion and host manipulation. For example, the plant natriuretic peptide-like peptide present in the citrus pathogen *Xanthomonas axonopodis* pv. *citri* functions as an antagonist of abscisic acid, while inducing responses similar to those induced by cytokinin [52]. This suggests that biotrophic pathogens can use plant-like hormones to modulate the host cellular environment, especially host metabolism, and that such modulations weaken host defenses. Molecular mimicry has also been reported in plant-parasitic nematodes. The CLAVATA3/ESR peptides in the cyst nematodes *Heterodera schachtii* and *Globodera rostochiensis* may play molecular mimetic roles in the regulation of certain root meristematic cells that are essential for the establishment of feeding sites in the host, a critical step in the process of nematode parasitism in plants [53–55]. In this study, we showed that these three proteins caused cell death when they were transiently expressed in tobacco plants. Water deficiency after PWN infection is a key factor in tree mortality [56]. During the process of tree death, a hypersensitive reaction is accompanied by the leakage of abnormal oleophilic substances from parenchymal cells [56]. It has been hypothesized that these leaked substances cover many bordered pits, resulting in blockage of the water-conducting system and, finally, tree death. To determine whether the cell death we observed in this study resulted from molecular mimicry, it will be necessary to perform protein 3D reconstruction of candidate proteins and deep sequence analysis focusing on shared domains. Moreover, it will also be useful to identify molecular interactors and determine the temporal and spatial patterns of molecular interactions. The peak of *Bx-TH1* gene expression occurs earlier than the gene expression of thaumatin-like protein in the stems and needles of pines [57]. The possible molecular mimetic role of the candidate proteins needs to be determined by *in vivo* identification of their interaction partners using analyses of protein-protein interactions, such as the yeast two-hybrid approach.

Bx-NA1 with SP and Bx-VA without SP also induced significant hypersensitive cell death compared to the GFP-only control (p < 0.05). Though the probabilities of Bx-NA1 and Bx-VA inducing cell death in *N*. *benthamiana* are lower than that of the three plant-like proteins, they seem to have a latent capability to induce cell death in plants. Bx-NA1 is a 459-amino-acid protein of unknown function. Since no ortholog has been found in other nematodes, it is likely a unique protein in *B*. *xylophilus* and may be important for the interaction with host pine trees. Venom allergen-like protein is a structurally conserved protein in many parasites, and it is suggested that venom allergen-like proteins of other plant-parasitic nematodes have important roles in infection [58,59]. *Bx-VA* is transcribed in pharyngeal gland cells, as shown by *in situ* hybridization [32]. Bx-VA is likely delivered into the host through the stylet, and it has a latent capability to induce cell death in host plants. The other five proteins examined in this study (Bx-AP, Bx-CP1, Bx-CP2, Bx-GST, and Bx-NA2) did not induce significant hypersensitive cell death relative to the GFP-only control. A hypersensitive cell death-like phenomena with low probability were observed in some of these constructs. This is probably due to a technical issue; some physical damage by syringe infiltration can cause cell death. Alternatively, some proteins that caused cell death with low probability may have weak effector functions. Since each plant has a different threshold of response to weak effectors and physical wounds, hypersensitive cell death-like symptoms may occur in some individuals. Although the clear function of these proteins in hypersensitive cell death was not determined, all of these proteins have putative SPs and are abundantly secreted by the PWN; hence, they have currently unknown roles in PWN infection. The ability of the PWN to avoid and/or tolerate plant defense responses may be a key element in the process of pine wilt [18], and these seven proteins may have functions in nematode avoidance or tolerance mechanisms.

We investigated the activities of 10 candidate molecules secreted by PWN using an *in planta* expression system. Five molecules induced hypersensitive cell death in *N*. *benthamiana*; interestingly, three of these had relatively high sequence similarity to plant proteins. Although these findings suggest a role for molecular mimicry by these candidate proteins, further investigation is necessary to prove this hypothesis.

## Materials and methods

### Nematode strains and culturing

We used the virulent Ka4 isolate of *B*. *xylophilus*, which was propagated on the BC-3 strain of the fungus *Botrytis cinerea*, grown at 25°C on malt extract agar plates (Difco Laboratories) containing 100 μg/mL chloramphenicol.

### Total RNA extraction from nematodes and cDNA synthesis

Total RNA was extracted from mixed-stage nematodes and purified using the RNeasy Mini Kit (QIAGEN), in accordance with the manufacturer’s protocol. Reverse transcription was performed with 1 μg of total RNA using the PrimeScript™ II 1^st^ strand cDNA Synthesis Kit (Takara Bio Inc.), in accordance with the manufacturer’s protocol. The transcribed cDNA was adjusted to a concentration of 200 ng/μL and subjected to PCR amplification.

### Gateway plasmid construction

The candidate proteins are listed in Table 1. All sequences were PCR amplified from cDNA extracted from *B*. *xylophilus*. All PCR assays were performed with Takara PrimeSTAR GXL DNA polymerase, in accordance with the manufacturer’s protocol (Takara Bio Inc.). The PCR components were as follows: 10 μL 5× Prime STAR GXL buffer, 4 μL dNTP Mixture (2.5 mM each), 1 μL forward primer (10 μM), 1 μL reverse primer (10 μM), 1 μL Prime STAR GXL Polymerase, 1 μL cDNA template (200 ng/μL), and 32 μL H_2_O. The cycling conditions were as follows: 35 cycles at 98°C for 10 s, 55°C for 15 s, and 68°C for 60 s/kb. The primers were designed using Primer 3 Plus software (http://www.bioinformatics.nl/cgi-bin/primer3plus/primer3plus.cgi). The presence of SPs in the associated genes was determined with the SignalP version 4.1 server. In this study, we prepared two constructs for each protein (with and without a putative SP) because the actual functions of the putative SPs are unknown and the localization or function may change in the presence or absence of the SP. The primers are listed in S1 Table.

Gateway-expression vectors were constructed with a BP-reaction, using the Donor vector pDONR207, and with an LR-reaction, using the Destination vector pUBC-GFP-dest, in accordance with the manufacturer’s specifications (Invitrogen). Gene sequences were determined by Sanger sequence analysis. The target genes were amplified and the stop codon was removed by PCR using primers containing the attB1 and attB2 sites. The products were cloned into the pDONR 207 entry vector, and then subcloned into the pUBC-GFP-Dest vector to generate a C-terminal GFP fusion. The primers used in this analysis are listed in S2 Table.

### Tobacco plant culture and transformation

*Nicotiana benthamiana* plants were grown in a growth chamber at 20–30°C under a 16 h-light/8 h-dark cycle. For all assays, the sixth to seventh leaves of 4–6-week-old specimens were used. Expression vectors harboring each gene were transformed into *Agrobacterium tumefaciens* strain C58 by electroporation. *Agrobacterium tumefaciens* bacteria harboring expression vectors were grown in Yeast Extract Beef media containing 100 mg/L spectinomycin, 50 mg/L carbenicillin, and 25 mg/L rifampicin. Cultures of *A*. *tumefaciens* suppressor strain P19 were grown at 28°C in Yeast Extract Beef media containing 50 mg/L kanamycin to an optical density at 600 nm of 0.5–0.7. Cells were harvested by centrifugation for 10 min at 3500 rpm, then re-suspended to a final optical density at 600 nm of 0.3 in 10 mM MES buffer with 10 mM MgCl_2_ and 150 μM acetosyringone. *A*. *tumefaciens* strains were mixed in roughly equimolar quantities; a volume of the P19 suppressor strain was then added, corresponding to approximately 25% of the total volume [60]. The *A*. *tumefaciens* suspension was manually infiltrated into *N*. *benthamiana* leaves using a 1-mL syringe. Symptom development was monitored visually for 10 days after infiltration and categorized visually into two classes: cell death or no cell death [61]. The proportions of spots of cell death were analyzed using Fisher’s exact test (** p < 0.01, * p < 0.05).

For co-localization analysis, the mCherry-labeled endoplasmic reticulum marker ER-rk CD3–959 was included for co-transformation [62]. The third or fourth leaf of 3-week-old *N*. *benthamiana* plants was agro-infiltrated with GFP or GFP fusion pathogenic candidate proteins with p19 and ER-rk CD3-959 in a 1:1:2 ratio. GFP or mCherry fluorescence in epidermal cells of the abaxial leaf side was assessed 3 days post infiltration using an LSM 880 laser scanning microscope fitted with a 40× objective lens (Carl Zeiss). Pieces of the infiltrated leaf were sampled randomly from the infected area and mounted in 400 mM sucrose for microscopic observation.

### *In situ* hybridization

*In situ* hybridization largely followed the procedures of Espada et al. [32]. The gene sequences of *Bx-TH1*, *Bx-TH2*, and *Bx-CPI* were PCR amplified from cDNA extracted from *B*. *xylophilus*. In the first round of PCR, each fragment was amplified with Takara PrimeSTAR GXL DNA polymerase, in accordance with the protocol described above in the section “Gateway plasmid construction.” The primers were designed using Primer 3 Plus software; these primers are listed in S3 Table. The resulting product was used as a template to synthesize ssDNA probes by linear PCR in a 30-μL reaction mixture (2.25 μL digoxigenin–DNA labeling mix [Roche Diagnostics], 6 μL 10 μM each reverse primer, 6 μL PCR buffer, 0.6 μL Prime STAR GXL Polymerase, 2.25 μL template, and 12.9 μL H_2_O). The cycling conditions were as follows: 95°C for 2 s, followed by 35 cycles of 95°C for 15 s, 55°C for 30 s, and 72°C for 90 s. The quality and yield of the reaction were tested by electrophoresis in a 1.5% agarose gel. The remaining reaction volume was boiled for 10 min and immediately quenched on ice to denature the ssDNA probe.

Adult *B*. *xylophilus* nematodes were fixed for 18 h at 4°C in 4 mL of 2% paraformaldehyde in M9 buffer, followed by 4 h in fixative at room temperature. The fixed nematodes were centrifuged and resuspended in 0.2% paraformaldehyde in M9 buffer, then chopped with a needle into 2–5 fragments on a clean microscope slide. The nematode sections were washed twice in 1 mL M9 buffer, then incubated at room temperature for 45 min in 0.5 mg/mL proteinase K (Life Technologies) in 500 μL M9 buffer. After nematodes had been washed in 1 mL of M9 buffer, they were pelleted, frozen at –20°C for 15 min, then incubated for 30 s in 1 mL of cold methanol at –20°C, followed by 1 mL of cold acetone for 1 min. After nematode sections had been pelleted at 13,000 rpm, they were rehydrated in distilled water.

The hybridization buffer contained 50% deionized formamide, 20% 20× SSC buffer, 1% 1× blocking reagent (10× blocking reagent: 10× maleic acid: diethyl pyrocarbonate-H_2_O at a 10:9:81 ratio), 2% sodium dodecyl sulfate, 1% 100× Denhardt’s solution, 1 mM ethylenediaminetetraacetic acid, 0.2 mg/mL fish sperm DNA, and 0.15 mg/mL yeast tRNA. The rehydrated nematode sections were washed in 500 μL of hybridization buffer, then pre-hybridized in 150 μL of hybridization buffer at 50°C for 15 min with rotation. Pre-hybridized nematode sections were added to 20 μL of DNA-digoxigenin probe. The hybridization process was allowed to proceed overnight at 50°C with rotation. The nematode sections were washed three times and incubated for 15 min with 100 μL of 4× SSC (20% 20× SSC and 80% diethyl pyrocarbonate-H_2_O] at 50°C with rotation, washed three more times, and then incubated for 20 min with 100 μL of 0.1× SSC/0.1% sodium dodecyl sulfate at 50°C with rotation. The nematode sections were washed once in 100 μL of 1× maleic acid (10× maleic acid: diethyl pyrocarbonate-H_2_O at a 1:9 ratio) and incubated with rotation for 30 min with 100 μL of 1× blocking reagent at room temperature. The nematodes were then labeled for 2 h with 100 μL of alkaline phosphatase-conjugated anti-digoxigenin antibody fragments that had been diluted 1:500 in blocking reagent. After the nematodes had been washed three more times and incubated with rotation for 15 min in 100 μL of 1× digoxigenin washing buffer (10× digoxigenin washing buffer: diethyl pyrocarbonate-H_2_O at a 1:9 ratio], they were stained overnight at 4°C in 100 μL of detection buffer with 0.34 μL of nitroblue tetrazolium and 0.35 μL of X-phosphatase. Staining was terminated by two washes in diethyl pyrocarbonate-H_2_O with 0.1% Tween 20. The nematodes were observed with differential interference contrast microscopy.

### Western blotting

Using a pestle and mortar, 1-cm^2^ portions of fresh or frozen *N*. *benthamiana* leaves were thoroughly ground in 40 μL of protein extraction buffer (4 μL 1 M Tris HCl, 8 μL glycerol, 28 μL H_2_O, and 1.6 mg SPS). The ground tissue was centrifuged for 10 min at 12,000 rpm to pellet the cellular debris. The supernatant was transferred to a new tube and stored at −80°C. Each protein extract was mixed in the same volume of 2× Laemmli sample buffer (Bio-Rad) with 2-mercaptoethanol (Bio-Rad) and heated at 95°C for 5 min. Ten microliters of the resulting products were loaded on a TGX Fast Cast gel (Bio-Rad) run at a constant voltage of 200 V for 30 min. Precision Plus Protein™ WesternC™ Standards (Bio-Rad) was used as a size marker. The activated gel was transferred to an Immun-Blot polyvinylidene fluoride membrane (Bio-Rad) using the Trans-Blot Turbo Transfer System (Bio-Rad) with Trans-Blot Turbo Midi Transfer Packs. The membrane was washed for 2 min with Tris-buffered saline with 0.05% Tween 20 (TBST), then blocked for 1 h in TBST with 1% skim milk powder. After the membrane had been blocked, it was washed three times in TBST for 5 min each. A rabbit polyclonal anti-GFP antibody (MBL, diluted 1:10,000) was combined in TBST buffer with 1% skim milk powder and incubated with the membrane for 1 h. After incubation with the primary antibody, the membrane was washed three times in TBST for 5 min each. A polyclonal anti-IgG-HRP antibody (secondary antibody, diluted 1:10,000) was mixed in TBST with 1% of skim milk powder and incubated with the membrane for 1 h, in combination with Precision Protein Steptactim-HRP conjugated diluted 1:50,000 (Bio-Rad). After the membrane had been incubated with the secondary antibody, it was washed three times in TBST for 5 min each. The membrane was then incubated for 5 min with Western ECL Substrate (Bio-Rad). The relative intensity of each band was compared using an Image Quant LAS 4000 biomolecular imager (Fujifilm).

## Supporting information

S1 Fig The reproducibility of mRNA localization of plant-like proteins in nematodes. Localization of *Bx-TH1* (A) and *Bx-CPI* (B) shown by in situ hybridization.
(TIF)

S1 Table List of primers used in the leaf-disk assay. (XLSX)

S2 Table List of primers used in Gateway vector construction. (XLSX)

S3 Table List of primers used in in situ hybridization. (XLSX)

S1 Raw image (TIF)

## References

[1] Kiyohara T, Tokushige Y. Inoculation experiments of a nematode, *Bursaphelenchus* sp., onto pine trees. J Jpn For Soc. 1971 7; 53(7):210–218.

[2] Yi CK, Byun BH, Park JD, Yang SI, Chang KH. First finding of the pine wood nematode, *Bursaphelenchus xylophilus* (Steiner et Buhrer) nickle and its insect vector in Korea. Res Rep For Res Inst Seoul. 1989; 38:141–149.

[3] Dwinell LD. The pine wood nematode: regulation and mitigation. Annu Rev Phytopathol. 1997 9; 35:153–166. 10.1146/annurev.phyto.35.1.153 15012519

[4] Han H, Chung Y-J, Shin S-C. First report of pine wilt disease on *Pinus koraiensis* in Korea. Plant Dis. 2008 8; 92(8):1251. 10.1094/PDIS-92-8-1251A 30769465

[5] Burgermeister W, Braasch H, Sousa E, Penas AC, Mota M, Metge K, et al First report of *Bursaphelenchus xylophilus* in portugal and in Europe. Nematology. 1999 1; 1(7):727–734.

[6] Abelleira A, Picoaga A, Mansilla JP, Aguin O. Detection of *Bursaphelenchus xylophilus*, causal agent of pine wilt disease on *Pinus pinaster* in northwestern Spain. Plant Dis. 2011 3; 95(6):77656. 10.1094/PDIS-12-10-0902 30731923

[7] Mamiya Y, Enda N. Transmission of *Bursaphelenchus lignicolus* (nematoda: Aphelenchoididae) by *Monochamus alternatus* (coleoptera: Cerambycidae). Nematologica. 1972 1; 18(2):159–162.

[8] Morimoto K, Iwasaki A. Role of *Monochamus alternatus* (coleoptera: Cerambycidae) as a vector of *Bursaphelenchus lignicolus* (nematoda: Aphelenchoididae). J Jpn For Soc. 1972; 54(6):177–183.

[9] Fukuda K. Physiological process of the symptom development and resistance mechanism in pine wilt disease. J For Res. 1997 8; 2(3):171–181.

[10] Yamada T. Biochemical responses in pine trees affected by pine wilt disease. Zhao BG, Futai K, Sutherland JR, Takeuchi Y, editors. Pine wilt disease. Tokyo: Springer Japan; 2008 p. 223–234.

[11] Myers RF. Pathogenesis in pine wilt caused by pine wood nematode, *Bursaphelenchus xylophilus*. J Nematol. 1988 4; 20(2):236–244. 19290207 PMC2618809

[12] Futai K. Pine wilt is an epidemic disease in forests: notes on the interrelationship of forest microbes. Tokyo: Bun-ichi sogo shyuppan; 2003.

[13] Hirao T, Fukatsu E, Watanabe A. Characterization of resistance to pine wood nematode infection in *Pinus thunbergiiusing* suppression subtractive hybridization. BMC Plant Biol. 2012 1; 12(1):13. 22272988 10.1186/1471-2229-12-13 PMC3398268

[14] Shinya R, Morisaka H, Takeuchi Y, Futai K, Ueda M. Making headway in understanding pine wilt disease: what do we perceive in the postgenomic era? J Biosci Bioeng. 2013 7; 116(1):1–8. 10.1016/j.jbiosc.2013.01.003 23474098

[15] Vicente CSL, Ikuyo Y, Mota M, Hasegawa K. Pinewood nematode-associated bacteria contribute to oxidative stress resistance of *Bursaphelenchus xylophilus*. BMC Microbiol. 2013 12; 13(1):299 10.1186/1471-2180-13-299 24365493 PMC3880045

[16] Vicente CSL, Ikuyo Y, Shinya R, Mota M, Hasegawa K. Catalases induction in high virulence pinewood nematode *Bursaphelenchus xylophilus* under hydrogen peroxide-induced stress. PLoS One. 2015 4; 10(4):e0123839 10.1371/journal.pone.0123839 25894519 PMC4404050

[17] Li Z, Zhang Q, Zhou X. A 2-Cys peroxiredoxin in response to oxidative stress in the pine wood nematode, *Bursaphelenchus xylophilus*. Sci Rep. 2016 6; 6:27438 10.1038/srep27438 27271000 PMC4895224

[18] Shinya R, Morisaka H, Kikuchi T, Takeuchi Y, Ueda M, Futai K. Secretome analysis of the pine wood nematode *Bursaphelenchus xylophilus* reveals the tangled roots of parasitism and its potential for molecular mimicry. PLoS One. 2013 6; 8(6):e67377 10.1371/journal.pone.0067377 23805310 PMC3689755

[19] Odani K, Sasaki S, Nishiyama Y, Yamamoto N. Early symptom development of the pine wilt disease by hydrolytic enzymes produced by the pine wood nematodes. J Jpn For Soc. 1985 9; 67(9):366–372.

[20] Kikuchi T, Jones JT, Aikawa T, Kosaka H, Ogura N. A family of glycosyl hydrolase family 45 cellulases from the pine wood nematode *Bursaphelenchus xylophilus*. FEBS Lett. 2004 8; 572(1–3):201–205. 10.1016/j.febslet.2004.07.039 15304348

[21] Kikuchi T, Shibuya H, Aikawa T, Jones JT. Cloning and characterization of pectate lyases expressed in the esophageal gland of the pine wood nematode *Bursaphelenchus xylophilus*. Mol Plant-Microbe Interact. 2006 3; 19(3):280–287. 10.1094/MPMI-19-0280 16570658

[22] Kikuchi T, Aikawa T, Kosaka H, Pritchard L, Ogura N, Jones JT. Expressed sequence tag (EST) analysis of the pine wood nematode *Bursaphelenchus xylophilus* and *B*. *mucronatus*. Mol Biochem Parasitol. 2007 9;155(1):9–17. 10.1016/j.molbiopara.2007.05.002 17560668

[23] Kikuchi T, Cotton JA, Dalzell JJ, Hasegawa K, Kanzaki N, McVeigh P, et al Genomic insights into the origin of parasitism in the emerging plant pathogen *Bursaphelenchus xylophilus*. PLoS Pathog. 2011 9; 7(9):e1002219 10.1371/journal.ppat.1002219 21909270 PMC3164644

[24] Zhang Q, Bai G, Yang W, Li H, Xiong H. Pathogenic cellulase assay of pine wilt disease and immunological localization. Biosci. Biotechnol. Biochem. 2006 11; 70(11):2727–2732. 10.1271/bbb.60330 17090937

[25] Jones JT, Moens M, Mota M, Li H, Kikuchi T. *Bursaphelenchus xylophilus*: opportunities in comparative genomics and molecular host-parasite interactions. Mol Plant Pathol. 2008 5; 9(3):357–368. 10.1111/j.1364-3703.2007.00461.x 18705876 PMC6640334

[26] Lin S, Jian H, Zhao H, Yang D, Liu Q. Cloning and characterization of a venom allergen-like protein gene cluster from the pinewood nematode *Bursaphelenchus xylophilus*. Exp Parasitol. 2011 2; 127(2):440–447. 10.1016/j.exppara.2010.10.013 20971105

[27] Kang JS, Koh YH, Moon YS, Lee SH. Molecular properties of a venom allergen-like protein suggest a parasitic function in the pinewood nematode *Bursaphelenchus xylophilus*. Int J Parasitol. 2012 1; 42(1):63–70. 10.1016/j.ijpara.2011.10.006 22142561

[28] Fire A, Xu S, Montgomery MK, Kostas SA, Driver SE, Mello CC. Potent and specific genetic interference by double-stranded RNA in *Caenorhabditis elegans*. Nature. 1998 2; 391(6669):806–811. 10.1038/35888 9486653

[29] Friedland AE, Tzur YB, Esvelt KM, Colaiácovo MP, Church GM, Calarco JA. Heritable genome editing in *C*. *elegans* via a CRISPR-Cas9 system. Nat Methods. 2013 8; 10(8):741–743. 10.1038/nmeth.2532 PMC3822328 23817069

[30] Mello C, Fire A. DNA transformation. Methods Cell Biol. 1995 4; 48:451–482. 8531738

[31] Park J-E, Lee KY, Lee S-J, Oh W-S, Jeong P-Y, Woo T, et al The efficiency of RNA interference in *Bursaphelenchus xylophilus*. Mol Cells. 2008 7; 26(1):81–86. 18525237

[32] Espada M, Silva AC, Akker SE van den, Cock PJA, Mota M, Jones JT. Identification and characterization of parasitism genes from the pinewood nematode *Bursaphelenchus xylophilus* reveals a multilayered detoxification strategy. Mol Plant Pathol. 2016 2;17(2):286–295. 10.1111/mpp.12280 25981957 PMC6638532

[33] Jayaraj J, Muthukrishnan S, Liang GH, Velazhahan R. Jasmonic acid and salicylic acid induce accumulation of β-1,3-glucanase and thaumatin-like proteins in wheat and enhance resistance against *Stagonospora nodorum*. Biologia Plantarum. 2004 9; 48(3):425–430.

[34] Futamura N, Tani N, Tsumura Y, Nakajima N, Sakaguchi M, Shinohara K. Characterization of genes for novel thaumatin-like proteins in *Cryptomeria japonica*. Tree Physiol. 2006 1; 26(1):51–62. 10.1093/treephys/26.1.51 16203714

[35] Kitajima S, Sato F. Plant pathogenesis-related proteins: molecular mechanisms of gene expression and protein function. J Biochem (Tokyo). 1999 1; 125(1):1–8. 10.1093/oxfordjournals.jbchem.a022244 9880788

[36] Brandazza A, Angeli S, Tegoni M, Cambillau C, Pelosi P. Plant stress proteins of the thaumatin-like family discovered in animals. FEBS Lett. 2004 8; 572(1–3):3–7. 10.1016/j.febslet.2004.07.003 15304314

[37] Grenier J, Potvin C, Asselin A. Some fungi express β-1,3-glucanases similar to thaumatin-like proteins. Mycologia. 2000 9; 92(5):841–848.

[38] Sakamoto Y, Watanabe H, Nagai M, Nakade K, Takahashi M, Sato T. Lentinula edodes tlg1 encodes a thaumatin-like protein that is involved in lentinan degradation and fruiting body senescence. Plant Physiol. 2006 6; 141(2):793–801. 10.1104/pp.106.076679 16648221 PMC1475445

[39] Wang J., Han S., Li Y., Deng X., Zhang X. Cloning of TLP-1 gene and prediction of TLP-1 protein structure of *Bursaphelenchus xylophilus*. J. Sichuan Agri. Univ. 2014 9; 32:305–310.

[40] Meng F, Li Y, Liu Z, Wang X, Feng Y, Zhang W, et al Potential molecular mimicry proteins responsive to α-pinene in *Bursaphelenchus xylophilus*. Int J Mol Sci. 2020 2; 21(3). Available from: https://www.ncbi.nlm.nih.gov/pmc/articles/PMC7037625/. 10.3390/ijms21030982 32024175 PMC7037625

[41] Chen WP, Chen PD, Liu DJ, Kynast R, Friebe B, Velazhahan R, et al Development of wheat scab symptoms is delayed in transgenic wheat plants that constitutively express a rice thaumatin-like protein gene. Theor Appl Genet. 1999 9; 99(5):755–760.

[42] Singh NK, Kumar KRR, Kumar D, Shukla P, Kirti PB. Characterization of a pathogen induced thaumatin-like protein gene AdTLP from *Arachis diogoi*, a wild peanut. PLoS One. 2013 12; 8(12):e83963 10.1371/journal.pone.0083963 24367621 PMC3868660

[43] El-kereamy A, El-sharkawy I, Ramamoorthy R, Taheri A, Errampalli D, Kumar P, et al *Prunus domestica* pathogenesis-related protein-5 activates the defense response pathway and enhances the resistance to fungal infection. PLoS One. 2011 3; 6(3):e17973 10.1371/journal.pone.0017973 21448276 PMC3063165

[44] Meng F, Li Y, Wang X, Feng Y, Liu Z, Zhang W, et al Thaumatin-like protein-1 gene (*Bx-tlp-1*) is associated with the pathogenicity of *Bursaphelenchus xylophilus*. Nematology. 2019 9; 109(11). 10.1094/PHYTO-03-19-0082-R 31573422

[45] Kuroda M, Kiyosaki T, Matsumoto I, Misaka T, Arai S, Abe K. Molecular cloning, characterization, and expression of wheat cystatins. Biosci Biotechnol Biochem. 2001 1; 65(1):22–28. 10.1271/bbb.65.22 11272836

[46] Arai S, Matsumoto I, Emori Y, Abe K. Plant seed cystatins and their target enzymes of endogenous and exogenous origin. J Agric Food Chem. 2002 10; 50(22):6612–6617. 10.1021/jf0201935 12381160

[47] Belenghi B, Acconcia F, Trovato M, Perazzolli M, Bocedi A, Polticelli F, et al AtCYS1, a cystatin from *Arabidopsis thaliana*, suppresses hypersensitive cell death. Eur J Biochem. 2003 6; 270(12):2593–2604. 10.1046/j.1432-1033.2003.03630.x 12787025

[48] Zhao Y, Botella MA, Subramanian L, Niu X, Nielsen SS, Bressan RA, et al Two wound-inducible soybean cysteine proteinase inhibitors have greater insect digestive proteinase inhibitory activities than a constitutive homolog. Plant Physiol. 1996 8; 111(4):1299–1306. 10.1104/pp.111.4.1299 8756506 PMC161012

[49] Pernas M, López-Solanilla E, Sánchez-Monge R, Salcedo G, Rodríguez-Palenzuela P. Antifungal activity of a plant cystatin. Plant Microbe Interact. 1999 7; 12(7):624–627.

[50] Xue Q, Wu X-Q. Characteristics and function of a novel cystatin gene in the pine wood nematode *Bursaphelenchus xylophilus*. Biol Open. 2019 9; 8(9). 10.1242/bio.042655 31511247 PMC6777362

[51] Santos CS, Pinheiro M, Silva AI, Egas C, Vasconcelos MW. Searching for resistance genes to *Bursaphelenchus xylophilus* using high throughput screening. BMC Genomics. 2012 11;13(1):599 10.1186/1471-2164-13-599 23134679 PMC3542250

[52] Garavaglia BS, Thomas L, Zimaro T, Gottig N, Daurelio LD, Ndimba B, et al A plant natriuretic peptide-like molecule of the pathogen *Xanthomonas axonopodis* pv. citricauses rapid changes in the proteome of its citrus host. BMC Plant Biology. 2010 3; 10(1):51 10.1186/1471-2229-10-51 20302677 PMC2923525

[53] Mitchum MG, Wang X, Wang J, Davis EL. Role of nematode peptides and other small molecules in plant parasitism. Annual Review of Phytopathology. 2012 5; 50(1):175–195. 10.1146/annurev-phyto-081211-173008 22578179

[54] Wang J, Replogle A, Hussey R, Baum T, Wang X, Davis EL, et al Identification of potential host plant mimics of CLAVATA3/ESR (CLE)-like peptides from the plant-parasitic nematode *Heterodera schachtii*. Molecular Plant Pathology. 2011 2; 12(2):177–186. 10.1111/j.1364-3703.2010.00660.x 21199567 PMC6640238

[55] Guo Y, Ni J, Denver R, Wang X, Clark SE. Mechanisms of molecular mimicry of plant CLE peptide ligands by the parasitic nematode *Globodera rostochiensis*. Plant Physiology. 2011 9; 157(1):476–484. 10.1104/pp.111.180554 21750229 PMC3165893

[56] Futai K. Pine wood nematode, *Bursaphelenchus xylophilus*. Annual Review of Phytopathology. 2013 5;51(1):61–83. 10.1146/annurev-phyto-081211-172910 23663004

[57] Meng F-L, Wang J, Wang X, Li Y-X, Zhang X-Y. Expression analysis of thaumatin-like proteins from *Bursaphelenchus xylophilus* and *Pinus massoniana*. Physiological and Molecular Plant Pathology. 2017 12; 100:178–184.

[58] Ding X, Shields J, Allen R, Hussey RS. Molecular cloning and characterisation of a venom allergen AG5-like cDNA from *Meloidogyne incognita*. International Journal for Parasitology. 2000 1; 30(1):77–81. 10.1016/s0020-7519(99)00165-4 10675748

[59] Lozano-Torres JL, Wilbers RHP, Warmerdam S, Finkers-Tomczak A, Diaz-Granados A, van Schaik CC, et al Apoplastic venom allergen-like proteins of cyst nematodes modulate the activation of basal plant innate immunity by cell surface receptors. PLoS Pathog. 2014 12; 10(12). Available from: https://www.ncbi.nlm.nih.gov/pmc/articles/PMC4263768/. 10.1371/journal.ppat.1004569 25500833 PMC4263768

[60] Atamian HS, Chaudhary R, Cin VD, Bao E, Girke T, Kaloshian I. In planta expression or delivery of potato aphid *Macrosiphum euphorbiae* effectors Me10 and Me23 enhances aphid fecundity. MPMI. 2013 1; 26(1):67–74. 10.1094/MPMI-06-12-0144-FI 23194342

[61] Kettles GJ, Bayon C, Canning G, Rudd JJ, Kanyuka K. Apoplastic recognition of multiple candidate effectors from the wheat pathogen *Zymoseptoria tritici* in the nonhost plant *Nicotiana benthamiana*. New Phytologist. 2017;213(1):338–350. 10.1111/nph.14215 27696417 PMC5132004

[62] Nelson BK, Cai X, Nebenführ A. A multicolored set of *in vivo* organelle markers for co-localization studies in Arabidopsis and other plants. The Plant Journal. 2007 8;51(6):1126–1136. 10.1111/j.1365-313X.2007.03212.x 17666025

