# Supplementary figures and images for: Thaumatin-like proteins and a cysteine protease inhibitor secreted by the pine wood nematode Bursaphelenchus xylophilus induce cell death in Nicotiana benthamiana
Source: PLoS One. 2020 Oct 30;15(10):e0241613. doi: 10.1371/journal.pone.0241613 (PMC7598465; doi:10.1371/journal.pone.0241613)

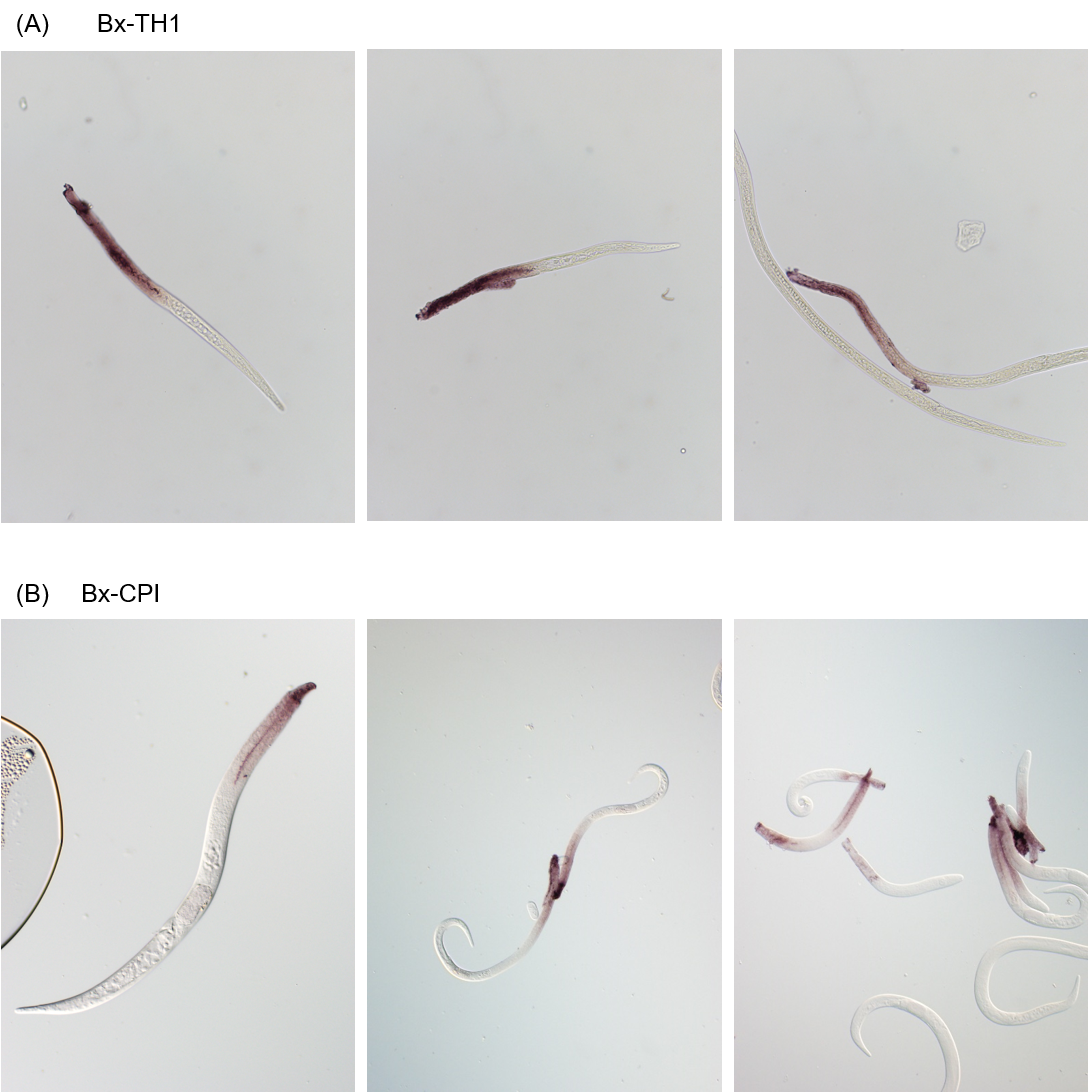

Supplement: S1 Fig — Localization of Bx-TH1 (A) and Bx-CPI (B) shown by in situ hybridization. (TIF) [file pone.0241613.s001.tif]

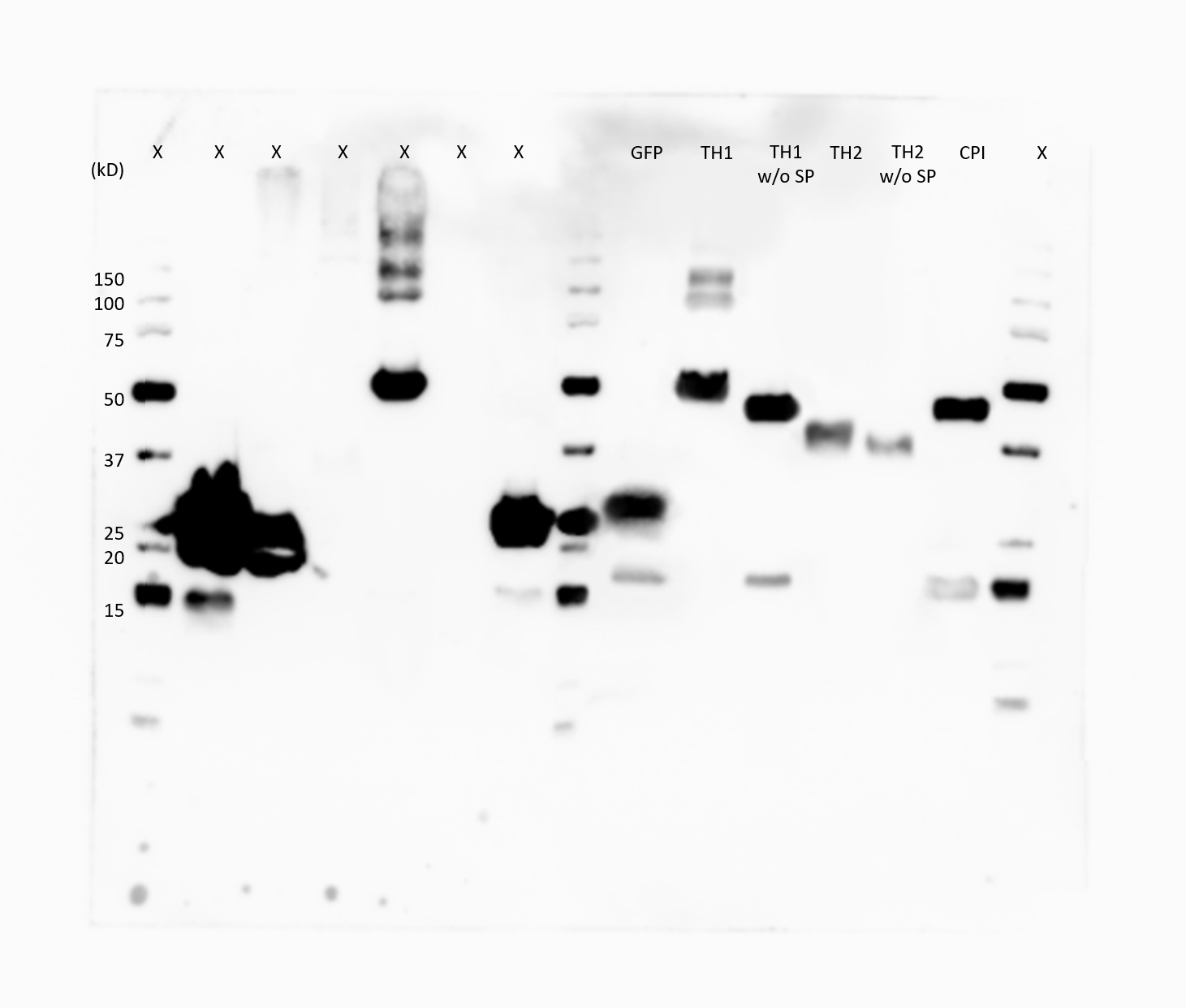

Supplement: S1 Raw image — (TIF) [file pone.0241613.s005.tif]
